# Neurobiology of language in autism: a systematic review of pediatric studies

**DOI:** 10.1007/s11682-026-01140-y

**Published:** 2026-03-25

**Authors:** Kate M. Witt, Amarie Carnett

**Affiliations:** 1 School of Education, Victoria University of Wellington, PO Box 600, Wellington, New Zealand; 2 School of Psychological and Social Sciences, University of Waikato, Hamilton, New Zealand

**Keywords:** Autism, Language, Communication, Neuroimaging

## Abstract

Language abilities are heterogeneous in autism spectrum disorder. Functional neuroimaging offers critical insights into the developmental trajectory of language in autism, with the potential to inform tailored supports. Unlike previous non-systematic or modality-specific reviews, this systematic review aimed to identify developmental trajectories in autistic children and elevated likelihood infants across functional neuroimaging modalities. Across the included 31 studies, EEG was the most common modality (39%), followed by fMRI (26%), MEG (26%), and fNIRS (9%). Most studies (77%) used passive awake tasks; around half employed simple speech stimuli (55%) while the remainder used complex speech (45%). There was an underrepresentation of minimally and nonspeaking autistic participants. fMRI and fNIRS studies found that elevated likelihood infants displayed hypoactivation in bilateral temporal regions, which did not consistently continue into mid-to-late childhood. EEG and MEG studies indicate that the timing and magnitude of auditory-perceptual and higher-order linguistic processing remained atypical (e.g., delayed latencies, larger amplitudes) from infancy to late childhood. Across modalities, findings were sometimes inconsistent, particularly when accounting for variability in language abilities. We highlight a strong need for caution in generalizing findings and argue that future research should prioritize more diverse samples to better elucidate the neural trajectory of language in autism.

**Supplementary Information:**

The online version contains supplementary material available at 10.1007/s11682-026-01140-y.

## Neurobiology of language in autism: a systematic review of pediatric studies

Autism spectrum disorder is characterized by difficulties in two primary domains: (1) social communication and interaction, and (2) restricted and repetitive behavior, interests, or activities (American Psychiatric Association, 2013). Although language impairment is no longer a core criterion in the DSM-5 (Bent et al., 2017), language and speech abilities are highly heterogeneous among autistic individuals, ranging from fluent speech to the absence of functional speech (i.e., nonspeaking) (Tager-Flusberg, 2015). Illustrating this variability, DSM-5 specifiers note that individuals requiring support (Level 1) may speak in full sentences, those requiring substantial support (Level 2) may use short phrases or simple sentences, and those requiring very substantial support (Level 3) may have very limited speech or rely primarily on nonverbal communication (American Psychiatric Association, 2013).

Moreover, language is included as a clinical descriptor in the DSM-5 to specify an autism diagnosis “with” or “without accompanying language impairment”, encompassing differences in receptive and expressive skills (American Psychiatric Association, 2013; Bent et al., 2017). Of note, there is also heterogeneity within the two specifiers; “with accompanying language impairment” can include lack of functional speech, speaking in single words, or phrase speech, and “without accompanying language impairment” can include speaking in full sentences or fluent speech (Rose et al., 2016). Recent estimates suggest that approximately 25–35% of autistic children and 25–30% of autistic adults are minimally verbal or nonspeaking (Rose et al., 2016; Tager-Flusberg & Kasari, 2013).

Early language abilities in autism strongly predict later language and communication outcomes. Autistic children with higher verbal ability often follow developmental trajectories similar to non-autistic peers, whereas autistic children with lower verbal abilities often show slower and/or flatter progress (Tek et al., 2014). While many minimally verbal children remain so into adulthood (Tager-Flusberg & Kasari, 2013), longitudinal studies highlight substantial variability: some autistic individuals acquire verbal language beyond early childhood (Markfeld et al., 2025). For example, Maltman et al. (2021) reported that 38% of autistic participants who were minimally verbal in early childhood became verbal in adulthood. Similarly, Brignell et al. (2018) reported that 19–30% of minimally verbal autistic children became verbal into adolescence and adulthood, underscoring the heterogeneity of language trajectories in autism.

This variability raises critical questions about the underlying mechanisms of language development in autism. Behavioral research has provided important insights, but understanding the neural underpinnings of these differences may help elucidate developmental trajectories and inform tailored interventions. Functional neuroimaging approaches, including electroencephalography (EEG), functional magnetic resonance imaging (fMRI), magnetoencephalography (MEG), positron emission tomography (PET), and functional near-infrared spectroscopy (fNIRS), offer promise for investigating the neural correlates of diverse language development in autistic children (Cermak et al., 2022; Sperdin & Schaer, 2016). However, multiple reviews have highlighted considerable variability in neural findings (Cermak et al., 2022; Gaudet et al., 2020; Herringshaw et al., 2016; Larson et al., 2023; Mody & Belliveau, 2013), which may reflect the potential heterogeneity of language in autism itself and/or methodological differences across studies. Specifically, this literature reflects a ranges of participant characteristics, experimental tasks, and differing neuroimaging modalities between studies (Groen et al., 2008), warranting further research into these factors.

Previous reviews have been limited by (1) non-systematic approaches, such as narrative or scoping reviews (e.g., Mody & Belliveau, 2013; Sperdin & Schaer, 2016), and/or (2) by focusing on only one or two imaging modalities (e.g., Cermak et al., 2022; Gaudet et al., 2020; Herringshaw et al., 2016; Larson et al., 2023). Additionally, prior reviews have not comprehensively examined how participant characteristics and study design contribute to observed neural differences. To address these gaps, the present review systematically synthesizes research on language development in autistic children across functional neuroimaging modalities, aiming to identify patterns and cross-modal connections that previous reviews may have missed. Our primary research question is: *What is the current evidence on the functional correlates of language development in autistic children?* Secondary questions include: (1) What are the characteristics of included participants?, (2) What experimental tasks are employed?, (3) Which neuroimaging modalities are used?, and (4) Which brain regions and/or temporal components are investigated?

## Methods

### Search strategy

We undertook this systematic literature review in accordance with the Preferred Reporting Items for Systematic reviews and Meta-Analysis (PRISMA) guidelines (Page et al., 2021). Comprehensive searches were conducted in *PsycINFO*,* PubMed*,* Medline*, and *Scopus* databases with the date range set from January 2014 to September 2024, providing a 10-year scope. Searches combined terms to describe autism and elevated likelihood of autism (i.e., auti*, pervasive developmental disorder, developmental disability, at risk for autism), language (i.e., language, communication, speech, voice, auditory), and functional neuroimaging modalities (i.e., electroencephalography, EEG, functional magnetic resonance, fMRI, functional near-infrared spectroscopy, fNIRS, magnetoencephalography, MEG, neuroimaging, positron-emission tomography, PET).

### Inclusion and exclusion

**Fig. 1:**
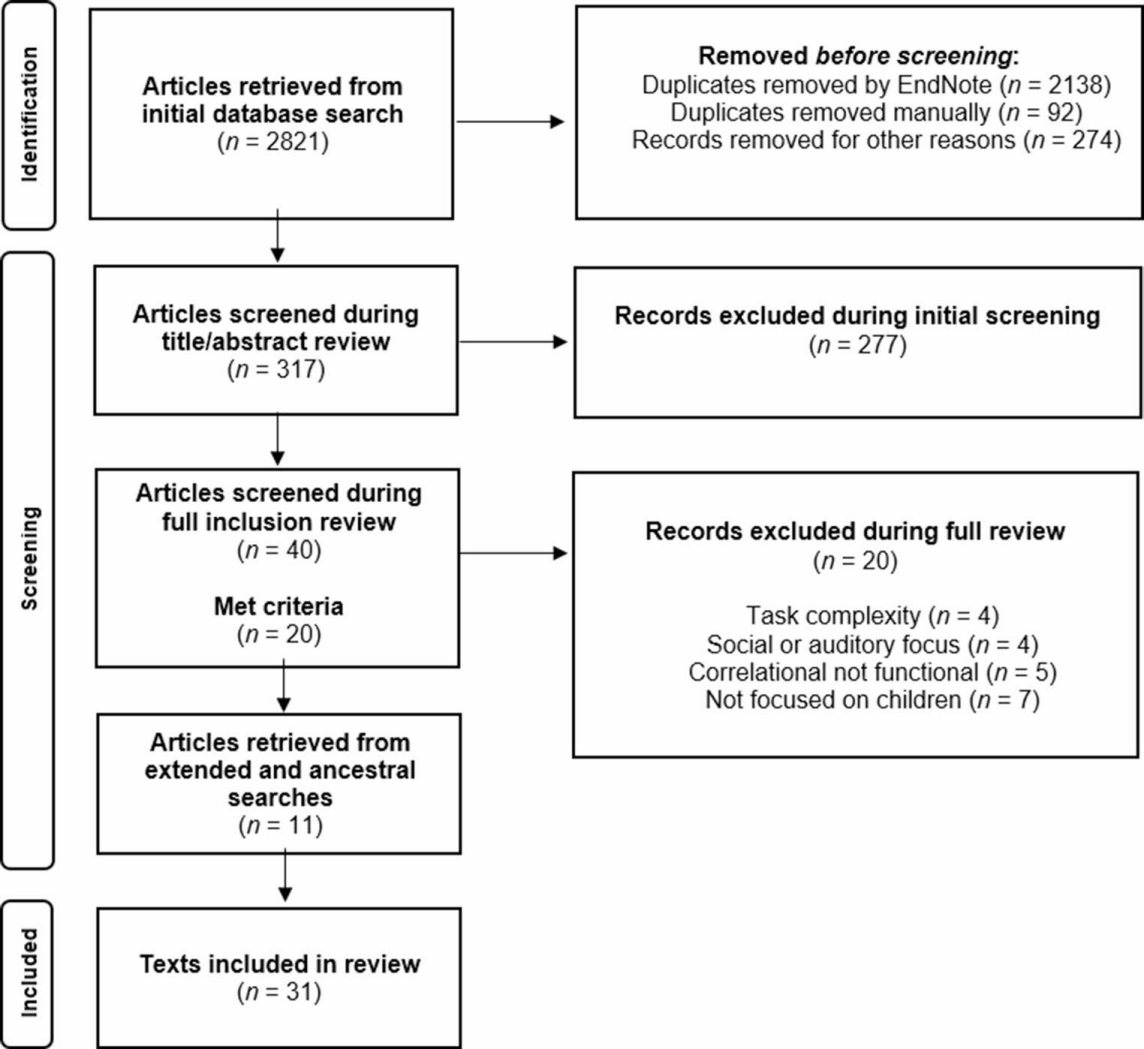
PRISMA diagram outlining search and screening procedure. Note. PRISMA = Preferred Reporting Items for Systematic Reviews and Meta-Analyses

Included studies met the following criteria: (a) were empirical, peer-reviewed research articles published in English; (b) included at least one participant with autism or elevated likelihood of autism; (c) involved at least one participant under 12 years of age; and (d) employed a functional neuroimaging measure (EEG, fMRI, fNIRS, MEG, or PET) (e) during a language task (e.g., phonological sound processing, sentence comprehension). Elevated likelihood of autism was defined as increased genetic risk, including younger siblings of autistic children and children with genetic disorders strongly associated with ASD (e.g., neurofibromatosis type 1, Fragile X syndrome; Al-Beltagi, 2021; Risch et al., 2014). Task stimuli could be presented passively or actively. Studies were excluded if they involved tasks only related to (a) social functioning (e.g., emotion recognition, face processing) or (b) sensory processing (e.g., abstract sounds or tones, auditory brain response), or they (c) employed a resting state measure. A PRISMA flow diagram detailing the number of articles included and excluded at each stage is shown in Fig. 1.

### Data extraction 

Each article was coded by both authors independently for the following variables: (a) number of participants, (b) participant characteristics (e.g., age, gender, ethnicity/race, socioeconomic status, autism specifiers), (c) neuroimaging modality, (d) neural measure of interest, (e) language task, and (f) core findings. We coded the number of participants, including comparison groups (e.g., non-autistic peers), and provided a narrative description of the neural measures, language tasks, core findings, and general conclusion.

### Quality evaluation method

We evaluated the studies using the Appraisal Tool for Cross-Sectional Studies (AXIS). This tool evaluates the quality of cross-sectional studies, including the study design, reporting quality, and risk of bias (Downes et al., 2016). It consists of 20 questions across five dimensions (introduction, methods, results, discussion, and other), with three potential answers (“yes”, “no”, “non-reported”/ “do not know”). The AXIS tool does not provide an overall quality score; therefore, we calculated the percentage of included items (“yes”) to determine how many of the 20 quality indicators were met. The wording of questions 13 (“Does the response rate raise concerns about non-response bias?”) and question 19 (“Were there any funding sources or conflicts of interest that may affect the authors’ interpretation of the results?”) is formulated opposite to the other indicators; therefore, a “no” on these two questions was indicative of meeting the quality indicators (Boluda-Verdú et al., 2022). AXIS rating decisions were made by both researchers, and a discussion of disagreements continued until consensus was achieved.

## Results

### Participant information

The 31 studies included a total of 1648 participants, including 905 autistic participants (55%) and 743 non-autistic participants (45%). The autistic groups had a median sample size of 22 participants (sample size ranged from 10 to 95 participants), and the non-autistic comparison groups had a median sample size of 20 participants (sample size ranged from 9 to 73 participants). Of the autistic participants, 83% (*n* = 752) had formal autism diagnoses, and 17% (*n* = 153) were classified with an elevated likelihood. Most autistic participants (*n* = 641; 71%) had average/above-average ranges on standardized measures of intelligence and/or expressive vocabulary, 12% (*n* = 106) had below average intelligence or a language impairment (i.e., low expressive or receptive vocabulary scores), and the remaining 17% (*n* = 158) did not have intelligence or language specifiers. Most studies (*n* = 29) included non-autistic or “low likelihood” comparison groups, and two studies included an additional comparison group of non-autistic participants with language or reading delays, accounting for 5% (*n* = 35 participants) of the non-autistic participants.

Across all participants, 24% were female, 56% were male, and 20% of participants’ genders were not reported. Participant ages ranged from > 1 month to 21 years (*proportional mean* = 6.63 years). Specifically, 33% of studies primarily included infants and toddler participants (1 month − 2 years), 16% included primarily young children (3–5 years), 45% included primarily school-aged children (6–11 years), and 6% included primarily adolescents (12–19 years). It is noted that the mean age is an estimate since not all studies reported exact participant ages. Only eight studies provided participants’ ethnicities/races and/or socioeconomic status (*n* = 3 ethnicities, *n* = 3 socioeconomic, *n* = 2 ethnicities + socioeconomic). Of the limited data provided, participants were predominantly white (ranging from 64.1 to 94.4% of participants) and had predominantly higher household income (medians reported were ~$75,000 USD).

### Experimental tasks

Most studies, 77% (*n* = 24), were conducted in the context of a passive awake task, which typically involved participants listening to speech stimuli while sitting still or engaging with something unrelated. Three of the passive studies were conducted while participants slept (Blasi et al., 2015; Liu et al., 2021; Lombardo et al., 2015). Four studies (Eigsti et al., 2016; Kovelman et al., 2015; Larson et al., 2022; O’Brien et al., 2023) used active tasks in which the participants responded to the speech stimuli. Around half of the studies’ (*n* = 17) stimuli consisted of speech sounds and syllables, while the remaining studies’ (*n* = 16) stimuli consisted of words or phrases. Most studies (*n* = 25) provided limited details about participants’ familiarity with the speech stimuli. Six studies mentioned using early words, basic nouns, or providing prior exposure to the stimuli.

### Quality ratings

**Fig. 2:**
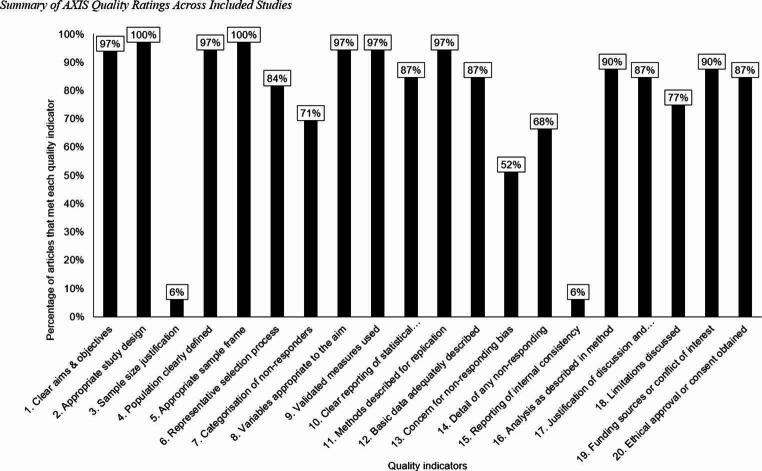
Summary of AXIS Quality Ratings Across Included Studies. Note. We assessed the study quality using the Appraisal Tool for Cross-Sectional Studies (AXIS; Downes et al., 2016). Each bar shows the proportion of studies that fulfilled a specific quality indicator. If the answer to questions 13 and/or 19 is “No”, this is considered meeting the quality indicator

Twenty studies (65%) met at least 70% (15 out of 20) or more of the quality indicators, suggesting good quality (see Supplementary Table 1; Boluda-Verdú et al., 2022). Across the quality indicators, at least 90% of the studies met the following indicators: (1) Clear aims and objectives; (2) Appropriate study design; 4. Population clearly defined; 5. Appropriate sample frame; 8. Variables appropriate to the aim; 9. Validated measures used; 11. Methods described for replication; 16. Analysis as described in method; and 19. Reported funding or conflict of interest. Other categories (6. Representative selection process; 7. Categorization of non-responders; 10. Clear reporting of statistical significance; 12. Basic data adequately described; 13; Concern for non-responding bias; 14. Detail of non-responding; 17. Justification of conclusion; 18. Limitations discussed; 20. Ethical approval or consent obtained) were included in at least half, but less than 90% of the studies. Only two studies (6%) included sample size justification (Q3; Begum-Ali et al., 2021; Dunham-Carr et al., 2023), and two reported information about internal validation (Q15; Cantiani et al., 2016; Demopoulos et al., 2023). See Fig. 2 for a summary of quality ratings.

### Cross-modal neural findings

Of the 31 articles, 39% (*n* = 12) used EEG, 26% used fMRI (*n* = 8), 26% used MEG (*n* = 8), and 9% (*n* = 3) used fNIRS as their functional modality. Although PET was included in our search terms, we did not find any studies using PET, which is possibly due to its higher cost, use of radiation, and age-related restrictions for radiation (Kowalewska et al., 2022; Tan et al., 2022). One study (Yau et al., 2015) employed two functional modalities, with MEG as the primary measure and EEG as the secondary measure. Tables 1, 2 and 3, and 4 detail the neuroimaging findings by modality, and the remaining results will discuss cross-modal findings. Given that language development follows an age-related trajectory, findings are best understood in relation to developmental stage.

**Table 1:** Descriptive Synthesis from EEG studies

Article	Participants	Neural measure	Task Description	Core findings	Conclusions
Begum-Ali et al., 2021	*N* = 59 (19 NF1; 40 TD) *Ages*: 5 and 10 m NF1 = higher autism risk	Examined amplitude of neural responses to sudden sound, repetitions, change in pitch (MMN), change in vowel category (MMN), and auditory discrimination between pitch and vowels in 50ms segments from 100 to 500 ms post stimulus. Collapsed across hemispheres to examine frontal and posterior regions.	*Passive (awake) task*: Auditory train task with each train composed of four consecutive 50-ms sounds with a 5-ms rise and fall time. The first three sounds in each train were repeated standards, and the fourth was a deviant. The sounds were presented for between 7 and 10 min, or until the infant became restless.	↓ change in amplitude to sudden sounds between 5 and 10 months for NF1 group compared to TD. ↓ slower response to repetition and pitch deviance (MMN) in frontal and posterior regions at 5 mnths but earlier responses at 10 mths in posterior regions in NF1 compared to TD. NF1 did not show a typical ↓ effect of age on repetition nor deviancy detection in posterior regions seen in TD. NF1 did not show ↑ response to pitch versus vowel stimuli seen in TD.	Infants with a higher risk for ASD showed slower neural detection of repetition or change in pitch and vowels and diminished developmental change of neural responses. Auditory responses did not relate to later language but were related to later ASD traits.
Cantiani et al., 2016	*N* = 20 (10 ASD NV/MV; 10 TD) *Ages*: 4:5 to 7:4 ASD; 4:1 to 7:8 yrs: m *NV/MV = non-verbal or minimally verbal	Examined time course of early sensory perception and higher-order linguistic processing via ERPs; basic visual processing (P1 at 150–200, 200–250 ms and PSW at 350–400, 400–450, 450–500 ms); basic auditory processing (auditory P1 at 100–120, 120–140, 140–160 ms); lexical-semantic processing (N400 at 350–500, 500–650, 650–800, 800–950 ms).	*Passive (awake) task*: A picture-word matching task with basic nouns. A total of 120 items were arranged in four pseudo-randomized blocks. Still pictures of animals or objects were presented for 2000 ms. A word that matched or mismatched the picture began after a 500-ms delay, while the picture remained on the screen. A second word, either semantically associated or non-associated with the first word was presented auditorily 500 ms after the offset of the first word. No behavioral response required.	↓ PSW amplitude across all three-time frames for ASD at occipital and central electrodes compared to TD. ↓ slower auditory P1 latency in ASD compared to TD with greater responses at later time frame (140–160 ms) compared to early time frames (100–120 and 120–140 ms). Average auditory P1 amplitude across time frames did not differ between groups. ↓ N400 amplitude (less positive) across all three-time frames for ASD compared to TD at occipital and central electrodes.	Atypical responses were seen at both early sensory perception and higher-order linguistic processing in the ASD group. Highlighted usefulness of passive paradigm for studying non-verbal or minimally verbal children.
Chen et al., 2021	*N* = 48 (24 ASD; 24 TD) *Ages*: 4 to 11 yrs	Examined neural phonetic coding of formant-exaggerated speech and nonspeech sounds via ERPs; P1 (50–90 ms), N1 (100–140 ms), N250 (180–300 ms), LNR (300–460 ms) at fronto-central region electrodes (F2, FCz, Fz, FC1, F1, C1, C2, FC2, Cz ).	*Passive (awake) task*: Two sessions (speech and nonspeech) were presented to each child with a counterbalanced sequence. In each session, alternating blocks of exaggerated and non-exaggerated sounds were presented for a total of 400 trials among 20 blocks, with each block containing 20 identical sounds. Block order was counterbalanced among the subjects. There were 800 experimental trials across two experimental sessions.	↑ P1 amplitude for speech stimuli than non-exaggerated vowel only for TD. ↑ N1 (more negative) amplitude for non-speech than speech condition for ASD, not TD. ↓ N250 (less negative) amplitude to speech for ASD compared to TD. Different LNR amplitude for non-exaggerated compared to formant-exaggerated vowel in both groups. ↑ P1 amplitude was associated with higher verbal IQ and chronological age predicted the P1 amplitude in ASD only.	Atypical processing of vowel exaggerated speech might be an early neural marker of risk in language development for children with ASD.
Dunham-Carr et al., 2023	*N* = 50 (25 autistic; 25 TD) *Ages*: 5.5 to 12.4 yrs	Examined ERP associated with processing distinctions between audiovisual and auditory-only speech; P2 at Cz electrode between 160–240 ms.	*Passive (awake) task*: Stimuli consisted of the consonant-vowel syllable “ba” as naturally spoken by an adult female speaker using a neutral facial expression. In the audiovisual condition, the corresponding auditory and visual stimuli were presented in synchrony.	↓ P2 amplitude for audiovisual compared to auditory-only speech in both groups. ↑ P2 suppression associated with ↑ receptive vocabulary across groups. Receptive and expressive vocabulary were positively correlated, which controlled for P2 amplitudes.	Efficiency of audiovisual speech processing may account for variance in vocabulary in autism.
Finch et al., 2017	*N* = 164 (73 LRC; 67 h; 23 ASD) *Ages*: 12 m Diagnosis outcome at 36 months.	Examined lateralization to speech sounds in infancy based on late low wave (LSW) ERP between 300–700 ms in left (FC1, FC5, C3, C5) and right (FC2, FC6, C4, C6) anterior regions.	*Passive (awake) task*: Stimuli consisted of a stream of three different consonant-vowel stimuli that were presented in a random order using a double-oddball procedure. A maximum of 600 stimuli were presented with varying interstimulus intervals.	↑ LSW amplitude in ASD than LR. HR did not differ from LR or ASD. ASD had reverse lateralization with ↑ LSW amplitude in left hemisphere than right whereas LR showed ↑ LSW amplitude in right.	Early atypical lateralization seen in infants later diagnosed with ASD; highlights potential early neural markers for ASD.
Finch et al., 2018	*N* = 74 (31 LR; 14 ASD; 13 h-A; 16 h-TD) *Ages*: 3 yrs HR-A = mild symptoms HR-TD = no symptoms	Examined ERPs associated with word processing. N200 between 200–350 ms and N350 between 350–500 ms in frontal (F3, F4, FC1, FC2, FC5, FC6) and temporo-parietal regions (P1, P2, P3, P4, P5, P6).	*Passive (awake) task*: Participants listened to a stream of two groups of concrete nouns: words acquired early, and words acquired late. Each noun was randomly presented a maximum of three times for up to 120 trials total.	No group differences in N200 or N350 at frontal sites. ↑ N200 amplitude (more negative) at temporo-parietal sites for late-acquired words in HR-A compared to ASD and LR. ↓ left N200 amplitude to late words correlated with more ADOS severity.	Atypical word processing in high-risk children with mild symptoms. Highlights importance of including language abilities and ASD diagnoses when examining neural differences in lexical processing.
Galilee et al., 2017	*N =* 28 (14 ASD (2 F, 12 M); 11 TD (2 F, 9 M); *Ages*: 4 to 6 yrs	Examined ERPs associated with the detection and categorization of speech versus non-speech; P150, N250, P350, N600 (frontal central region) and N330, P600 (temporal region).	*Passive (awake) task*: Novel paired repetition paradigm. Participants listened to paired sound stimuli (both speech, both non-speech, speech then non-speech or non-speech then speech). Speech sounds = syllables (ba, ga, da) and non-speech = non-phonetic correlates.	No group differences in P150, N250, P350. ASD only had left temporal N330 mismatch response for speech followed non-speech, not bilateral N330 seen in TD. ↓ temporal P600 and central N600 responses for mismatch with non-speech followed by speech in ASD.	ASD show atypical speech versus non-speech processing with no right hemisphere activation and decreased responses to mismatch.
Green et al., 2020	*N* = 25 (7 ASD + LI [2 F, 5 M]; 8 ASD – LI [1 F, 7 M]; 10 TD [3 F, 7 M]) *Ages*: 5 to 10 yrs LI = language impairment	Examined ERP associated with directing attention to novel sounds (mismatch negativity [MMN]) between 150–200 ms at frontocentral left (12, 13, 18, 19, 20, F3) and right (4, 5, 10, 112, 118 F4) electrodes.	*Passive (awake) task*: Auditory oddball task with speech (vowels) and pure tone sounds. One tone or vowel was presented 85% of the time (standard) and another 15% (deviant aka “oddball”). Participants watched silent video while listening to the stimuli.	TD and ASD + LI showed MMN response across hemisphere for tones while ASD - LI showed no MMN for tones. All 3 groups showed MMN across hemispheres for vowels. ↓ MMN latency (faster) for vowels in ASD + LI than ASD-LI and TD combined.	Consistent MMN for tones and speech in TD, but only consistent for speech in ASD groups. Faster speech processing for language impairment group could be due to increased connectivity in primary sensory cortices at the expense of connectivity to other areas.
Sandbank et al., 2017	*N* = 34 (34 pre-verbal ASD [7 F, 27 M]). *Ages*: 2 to 5 yrs *no TD group	Examined ERPs associated with distinguishing words from nonwords; negative component between 200-500ms at left temporal T3 region (44, 45, 46, 50, T7) and left parietal P3 region (42, 47, 52, 53, 59, 60).	*Passive (awake) task*: Participants listened to a recording of 10 words commonly learned early and 10 nonwords matched to duration and syllables. Stimuli were randomly presented at variable intervals (each word presented 3 times total). Participants watched silent video while listening to the stimuli. Later, there were ratings of whether the children understood the words.	↑ response to words than nonwords between 200-500ms at both T3 and P3, but none of the differences were significantly greater than zero. No correlations between receptive vocabulary scores or ASD severity and response at T3 or P3. Word-nonword differences in amplitude at T3 and the number of words understood predicted receptive vocabulary.	Typical neural responses between word and nonword were not seen. Understanding words seems to impact neural responses.
Seery et al., 2014	*N* = 80 (35 h [17 F, 18 M]; 45 LR [22 F, 23 M]). *Ages*: 9 m	Examined positive ERP at 150–300 ms (P150) associated with early language processing of speech stimuli. Electrodes of interest at frontal (F1, F2, F3, F4, F7, F8, AF3, AF4) and central (FC1, FC2, FC5, FC6, C3, C4, C5, C6).	*Passive (awake) task*: Participants listened to a recording of consonant-vowel stimuli in a double oddball paradigm. One consonant-vowel was presented 80% of the time (standard) and another randomly 10% of time. Participants could play with toys during task.	↑ P150 amplitude for standard speech repetitions at frontal electrodes in HR. No group difference in P150 for “oddball” speech stimuli. ↑ P150 at 9 months associated with higher language scores at 18 months in ASD.	Atypical processing of repetitive speech in HR infants. Atypical responses were associated with better language development, not impaired.
Tran et al., 2021	*N* = 63 (36 h [12 ASD concern, 24 no concern]; 27 LR [2 ASD concern, 25 no concern]). *Ages*: 3 m	Examined power and phase coherence at theta and alpha frequency bands within language networks across hemispheres (fronto-central & fronto-temporal). Electrodes of interest at frontal (F3, F4, F7, F8, F9, F10), temporal (T7, T8, T9, T10) and central (C3, C4).	*Passive (awake) task*: Participants listened to recordings of continuous stream of concatenated syllables that consisted of 4 trisyllabic pseudo-words. Infants sat in parents lap upright. Participants were tested at 18 months for ASD symptoms and word production.	↓ theta and alpha phase coherence at left fronto-central pair (F9-C3) for ASD-concern (trend, not significant). No group differences in theta nor alpha spectral power at any ROIs. Across groups, alpha coherence at F9-C3 at 3 months correlated with ↑ word production at 18 months.	Connectivity via phase coherence can be quantified as early as 3 months with reduced left fronto-central connectivity in ASD concern. Early atypical connectivity could be a marker for language development.
Yu et al., 2023	*N* = 46 (21 ASD-MV [2 F, 19 M]; 25 TD [5 F, 20 M]. *Ages*: 9 to 14 yrs *MV= minimally verbal	Examined ERPs associated with prosodic phonology processing (left electrodes F3/C3 and right F4/C4); P1 (sound detection) at 50-90ms, N1 (acoustic saliency, audibility, and consonant-vowel categorization) at 100-150ms, LNR for language at 300-800ms in fronto-central. General phase coherence for theta and delta bands.	*Passive (awake) task*: Mandarin-speaking participants listened to recordings of 10 nonsense disyllabic pseudowords, 10 nonnative words (English) matching syllabic structure, and non-speech (humming) stimuli. They watched muted videos during the task.	P1-N1 present in both groups for speech with later N1 in ASD. ↑ N1 for speech than non-speech in TD but ASD showed opposite with ↑ P1 and N1 for non-speech. ↑ theta and delta coherence for non-speech than speech in ASD. Both had larger LNR to speech than non-speech. LNR left lateralization for speech in TD but no lateralization in ASD.	Reduced neural specialization for abstract word-level prosodic phonology in ASD.

**Table 2:** Descriptive Synthesis from fMRI studies

Article	Participants	Neural measure	Task Description	Core findings	Conclusions
Abrams et al., 2019	*N* = 57 (21 ASD; 21 TD) *Ages*: 10.75 ASD; 10.32 TD	Examined clusters of activation via whole-brain analysis during each vocal condition. Functional connectivity during each vocal condition was examined in pre-defined voice processing ROIs. *Regions of interest*: left pSTS, right pSTS, left vmPFC, right vmPFC, left anterior insula, right VTA, NAc, left OFC, putamen, caudate, right amygdala, right HP, right FG.	*Passive (awake) task*: Randomized, rapid event-related design was used. Three auditory nonsense words, produced by three different speakers, were presented to the child participants. Three vocal conditions: mother’s voice, unfamiliar voices, and environmental sounds. Auditory stimuli were 956 ms in duration.	↓ right PP in STG to voices in ASD. Weaker difference between voices and environmental sounds in ASD. ↓ intercalcarine cortex, FG, PC, right posterior HP to mother’s voices in ASD. ↑ medial HG, right PP, mSTS, right rostral ACC, anterior insula, vmPFC associated with higher social communication scores in ASD. Multivariate connectivity patterns for mother’s voice can distinguish between ASD and TD group.	Reward system deficits for processing social stimulus (mother’s voice) in ASD group supporting social motivation theory. Activity in response to mother’s voice in reward, auditory, and voice selective regions predicts social communication abilities in ASD.
Blasi et al., 2015	*N* = 33 (15 h of ASD; 18 LR) *Ages*: >1 to 5 m	Examined significant clusters of activation via whole-brain analysis between groups and conditions: neutral voice versus non voice and sad voice versus neutral voice. *Regions of interest*: whole-brain, auditory cortex was only pre-defined region.	*Passive (natural sleep) task*: Infants were presented with three categories of adult non-speech vocalizations (emotionally neutral, emotionally positive, and emotionally negative) sounds and non-vocal familiar environmental sounds. A complete session comprised 32 blocks (8 in each stimulus category) lasting 16 min.	LR had ↑ activation in bilateral SMTG, SFG, MFG, and right CG for voices but HR only had ↑ activation in right IPL. LR showed preference for voice over non-vocal, but HR had preference for non-vocal over voices in left MTG and bilateral SFG and MFG. LR had ↑ activation than HR in right FG and left HP to sad than neutral voices. ↑ interaction scores were associated with ↓ activation to voice over non-vocal sounds in HR.	HR had atypical neural responses to human voice with and without emotional valence.
Eigsti et al., 2016	*N* = 59 (16 OO; 23 ASD-HF; 20 TD) *Ages*: 8 to 21 yrs *OO = “optimal outcome” HF = high functioning	Examined significant clusters of activation via whole-brain analysis between groups and conditions: high versus low imagery. *Regions of interest*: whole brain (not pre-specified in the method).	*Active task*: Stimuli consisted of short declarative statements displayed for up to 4s until a response was made. Participants made a true/false judgment about each statement via button press. Sessions included 12 low-imagery, 12 high-imagery, and 24 control trials presented in pseudo-random order.	↑ activation for OO group compared to TD and ASD in right SFG, right SMG, left preG/postG, right MFG, right and left STG, left IPL, left IMFG, and right and left anterior/posterior cerebellum (reflective of compensation). ↑ activation for OO and ASD group compared to TD in left MFG, posterior CG, left SMG, and right SMTG (reflective of residual ASD).	OO group had similar comprehension (behaviorally) to TD but had higher recruitment of language-related regions, reflecting neural compensation.
Larson et al., 2022	*N =* 55 (22 ASD; 14 LAD; 19 TD). *Across LAD & ASD groups 15 LI.* *Ages*: 8 to 21 yrs *LAD = loss of diagnosis LI = language impairment	Examined brain activation and lateralization across hemispheres in pre-determined language ROIs. *Region of interest*: Frontal cluster in 44, 45, 47 medial, posterior 47 rostral, anterior 47 rostral, 47 lateral, 47 s, 6 rostral, IFSp, IFSa, IFJa, and IFJp. Temporal cluster in STSda, STSdp, STSva, STSvp, TE1a, TE1m, TE1p, TE2a, and PHT. Semantic cluster in 44, 45, 55b, IFJa, 8 C, SLF, SCEF, 8BM, STSdp, STSvp, AIP, PFm, TE1p, PHT, and PBelt.	*Active task*: Participants read sentences and made a true/false value judgment via button press for the experimental condition and participants made a left/right button press for the control condition. High imagery (e.g., The number eight when rotated 90 degrees looks like a pair of eyeglasses) and low imagery tasks (e.g., Addition, subtraction, and multiplication are all math skills).	↑ participants in ASD and LAD met criteria for “structural language impairment criteria” than TD. ↑ left lateralization in frontal and temporal clusters for LI than LN (across ASD, LAD and TD). No difference in semantic cluster. ↑ left lateralization in frontal and temporal clusters in individuals with more communication difficulties and repetitive behaviors.	Language impairment is associated with unique neural specialization for language function, even without current ASD symptoms.
Liu et al., 2021	*N =* 43 (16 LR [9 F, 7 M]; 27 h [8 F, 19 M]). *Ages*: 9 m	Examined neural activation for speech conditions (stressed, unstressed, random) to identify neural correlates underlying word segmentation (whole-brain analysis and pre-determined ROIs). *Regions of interest*: IFG, preG, postG, SMA, GP, HG, HP, SMG, IPL, STG, SMTG, PHG, IOFC, mSTG, mSTG, thalamus, amygdala, putamen, caudate, insula.	*Passive (natural sleep) task*: Infants were exposed to 3 counterbalanced streams of nonsense speech presented in one continuous block. Transitional probabilities and prosodic cues were manipulated into 3 conditions (Stressed with transitional probabilities and prosodic cues; unstressed with transitional probabilities only; random syllables with no transitional probabilities or prosodic cues.)	Bilateral temporal cortices activation across groups during speech than silence. LR had ↑ activation in amygdala during stressed condition compared to HR. LR had ↑ activation in bilateral STG and left HG during stressed compared to unstressed, with ↑ difference between conditions compared to HR. ↑ activation in left STG and HG at 9 months positively correlated with communication scores at 36 months.	HR showed limited neural activity associated with prosodic processing and implicit language learning compared to LR. Early atypicality in language neural networks may predict language acquisition and altered developmental trajectories in at risk infants.
Lombardo et al., 2015	*N =* 103 (60 ASD [24 poor language outcomes, 36 good language outcome]; 19 LD; 24 TD). *Ages*: 12–48 m LD = language delayed (non-ASD)	Examined brain activation for language regions of interest and general auditory processing regions outside of speech and language areas. *Regions of interest*: A1 and bilateral frontal and temporal cortex language network (ROIs from Neurosynth detailed in their supplementary).	*Passive (natural sleep) task*: Pre-diagnosis infants were exposed to 3 types of stimuli (complex forward speech, simple forward speech, and backward speech). Simple speech was taken from a toddler’s book (1–2 syllable words), complex speech taken from poetic older children’s book (3 syllable words). At age of brain scanning, participants were not diagnosed but diagnosis and language outcome followed up until 3–4 years.	TD, ASD good, LD showed similar activation across left PP, PT, PO, HG, MTG, ITS, AG, SMG, IPS, pSTS, mSTS, and aSTS (language ROIs). ASD poor had ↓ activation in left PP, PT, HG, MTG, ITS, pSTS, mSTS, and aSTS compared to TD, ASD good, and LD. All groups showed similar activation for general auditory processing in A1. ↑ activation in thalamus, retrosplenial, and occipital cortex for forward compared to backward speech for TD only. Language ROIs activation positively correlated with language outcomes in LD and TD, but negatively correlated in ASD good and ASD poor. Combination of clinical measures and left STC activation predicted language outcome better than neural or clinical markers alone.	Before ASD diagnosis and language outcome is clear, distinct neural markers exist in language regions. Clinical measures and neural activation in language ROIs can predict outcomes.
O’Brien et al., 2023	*N =* 67 (28 TD [8 F, 20 M]; 23 ASD [5 F, 18 M]; 16 RD [7 F, 11 M]). *Ages*: 5 to 18 yrs *RD = reading disability	Examined brain activation for non-word repetition in 3 networks of interest and intersection of these networks. *Regions of interest*: Speech perception network, working memory network, and speech production network including cerebellum, SMA, STG (ROIs from Neurosynth detailed in their supplementary).	*Active task*: Nonword repetition to measure phonological working memory. Participants listened to non-words and repeated them back out loud. They were told the nonwords were from an “alien language” with a colorful alien image on the screen to prompt responses.	ASD and RD had ↓ repetition accuracy than TD. ASD no different to RD. ASD and TD showed activation clusters (repetition vs. rest) in bilateral STG and SMA. No group differences in speech perception nor working memory NOIs. ↑ SMA activation to longer than shorter words for TD, with ↑ SMA activation to shorter words than longer words for ASD (speech production network). SMA activation for TD correlated with ↑ repetition accuracy, but ASD showed no correlations.	Phonological working memory challenges in ASD might be due to differences in speech production networks (SMA), not speech perception.
Sharda et al., 2015	*N* = 44 (22 ASD (6 F, 16 M with 11 HF, 2 PDD-NOS, 9 LF); 22 TD (6 F, 16 M). *Ages*: 6 to 16 yrs *HF = high functioning *LF = low functioning	Examined neural activation for sound processing (sung versus spoken) via an event-related auditory paradigm. Also examined structural integrity of white matter tracts associated with language via DTI. *Regions of interest*: No pre-defined ROIs for fMRI. Major white matter tracts: bilateral ILF, bilateral SLF (dorsal, temporal), bilateral UF (DTI).	*Passive (awake) task*: Participants listened to recordings of 30 spoken words, 30 sung words and 15 piano tones. Spoken words matched the sung words, sung words matched notes to piano.	Both groups showed leftward activation in MTG, STG, and STS for sung-words and tones. ↓ left IFG activity for spoken-words in ASD. ↓ white matter integrity in left SLF which predicted left IFG activation for spoken-words in ASD. TD showed leftward lateralization while ASD showed rightward lateralization for spoken-words. ↑ left IFG activation associated with higher language scores in ASD.	Atypical activation and lateralization with disrupted fronto-temporal connectivity during spoken-word processing in ASD, but not song and tone processing. Suggests alternate mechanisms of speech and music processing in ASD.

*AG* angular gyrus, *ACC* anterior cingulate cortex, *AIP* anterior intra-parietal area, *aSTS* anterior superior temporal sulcus, *CG* cingulate gyrus, *FG* fusiform gyrus, *GP* global pallidus, *HG* Heschl’s gyrus, *HP* hippocampus, *IC* inferior colliculus, *IMFG* inferior and middle frontal gyri, *IFG* inferior frontal gyrus, *IFSa* interior frontal sulcus anterior, *IFSp* interior frontal sulcus posterior, *IFJa* interior frontal junction anterior, *IFJp* interior frontal junction posterior, *ILF* inferior longitudinal fasciculus, *IOFC* inferior orbitofrontal cortex, *IPL* inferior parietal lobe, *ITS* inferior temporal sulcus, *IPS* intraparietal sulcus, *LOC* lateral occipital cortex, *mSTS* medial/middle superior temporal sulcus, *MFG* middle frontal gyrus/premotor cortex, *MTG* middle temporal gyrus, *NAc* nucleus accumbens, *OFC* orbitofrontal cortex, *PBelt* Para-belt complex, *PHG *Parahippocampal Gyrus, *PFm* parietal area F medial, *PO* parietal operculum, *PP *planum polare, *PT *planum temporale, *pSTS *posterior superior temporal sulcus, *preG *precentral gyrus, *postG* postcentral gyrus, *PC *precuneus cortex, *A1 *primary auditory cortex, *SMTG* superior and middle temporal gyri, *SFG *superior frontal gyrus, *SLF* superior longitudinal fasciculus, *STC *superior temporal cortex, *STG *superior temporal gyrus, *STSda *superior temporal sulcus dorsal anterior, *STSdp *superior temporal sulcus dorsal posterior, *STSva* superior temporal sulcus ventral anterior, *STSvp* superior temporal sulcus ventral posterior, *SCEF* supplementary cingulate eye field, *SMA* supplementary motor area, *SMG* supramarginal gyrus, *TE1a* temporal area 1 anterior, *TE1m* temporal area 1 medial, *TE1p* temporal area 1 posterior, *TE2a* temporal area 2 anterior, *TPJ* temporal parietal junction, *UF* uncinate fasciculus, *vmPFC* ventromedial prefrontal cortex, *VTA* ventral tegmental area

**Table 3:** Descriptive Synthesis from fNIRS studies

Article	Participants	Neural measure	Task Description	Core findings	Conclusions
Edwards et al., 2017	*N* = 38 (21 h; 17 LR) *Ages*: 3 m	Examined strength of hemoglobin concentration (oxyHB) associated with structural regularities in speech. Four ROIs: left anterior (channels 1–4), left posterior (channels, 9–12), right posterior (channels 13–17) and right anterior (channels 20–24).	*Passive (awake) task*: The stimuli were comprised of repeating or non-repeating trisyllabic sequences. In repeating sequences, the second and third syllables were identical while in the non-repeating sequences, all syllables were different. Trials were presented in one of two pseudo-randomized orders Each child heard a maximum of 28 trials.	↑ oxyHB responses in left anterior for HR females compared to HR males and LR group for speech-like stimuli. ↑ oxyHB responses in right anterior for HR females compared to HR males and LR females for speech-like stimuli. ↓ oxyHB responses to repetitive speech-like stimuli for LR group and HR males, but not HR females.	Female infants at high-risk for ASD do not show typical decrease in neural response with repeated exposure to speech stimuli, which might reflect a neural endophenotype of language development or ASD specific to females.
Lai et al., 2024	*N =* 40 (20 ASD; 20 TD) *Ages*: 6.5 to 1.7 yrs	Examined hemispheric lateralization across bilateral temporal and frontal parietal lobes for processing linguistic auditory stimuli as a function of linguistic relevance.	*Passive (awake) task*: Four stimulus conditions (natural native speech, native speech with scrambled word order, nonnative speech, and music) were presented in a block trail design. Each condition was divided into nine 15-s segments, totaling 36 trials.	ASD had ↓ activation in left temporal cluster than TD across stimuli. Right temporal cluster did not differ between groups. ASD did not show left lateralization in temporal cluster across stimuli. ASD had ↑ right temporal activation for scrambled native speech while TD had ↓ left hemisphere activation as linguistic relevance decreased.	Atypical lateralization for spoken language in autistic children is associated with phonological aspects of speech processing.
Pecukonis et al., 2021	*N* = 32 (18 LR [9 F, 9 M]; 14 h* [7 F, 7 M]). *Ages*: 6 m	Examined strength of hemoglobin concentration (oxyHB) in response to speech across left anterior (channels 1–4), right anterior (channels 20–24), left posterior (channels 9–12), and right posterior (channels 13–17) regions.	*Passive (awake) task*: Participants listened to recordings of 2 types of speech (repetitive and random syllable sequences). Repetitive speech: presentation of 3 sequential syllables with last 2 being identical. Random speech: presentation of 3 sequential syllables (all syllables differed).	↑ activation for speech in bilateral anterior regions than posterior regions in LR, but no difference between ROIs for HR. HR had ↓ activation in bilateral anterior ROIs and ↑ activation in right posterior ROIs than LR.	LR infants show functional specialization for speech processing, not lateralization, by 6-months of age. HR infants do not display specialization for speech.

**Table 4:** Descriptive Synthesis from MEG studies

Article	Participants	Neural measure	Task Description	Core findings	Conclusions
Alho et al., 2023	*N* = 51 (25 ASD; 26 TD) *Ages*: 7 to 17 ASD and TD	Examined ERFs 0-1500ms after stimulus in pre-defined regions. Cerebro-cerebellar functional connectivity was measured via phase synchrony between cerebellar lobules and cerebral ROIs. *Regions of interest*: Cerebellum lobules (VI and VII) across both hemispheres. Auditory and language regions including STG/A1, MTG, SMG, M1, MFG, and IFG.	*Passive (awake) task*: The stimuli were presented binaurally. Each stimulus was presented 40 times in the paradigm (80 Speech, 80 Jabberwocky, and 80 Noise trials total). Participants watched a muted video of their choosing and were instructed to ignore the sound stimuli during the session. The paradigm was presented three times, lasting about 3 min. each.	↑ late ERFs (1000–1500 ms) for meaningless than meaningful condition in left temporal and parietal ROIs in ASD compared to TD. ↑ early ERFs (100–700 ms) for meaningful than meaningless condition in parietal ROIs in TD compared to ASD. No difference between meaningful and meaningless sentences in right lobule VI in ASD compared to TD. ↓ functional connectivity for meaningful condition between right lobule VI and MTG, M1, SMG, MFG, IFG, A1 in ASD. ↑ functional connectivity in meaningful condition between right lobule VI and left A1 in ASD.	Atypical functioning patterns in ASD were correlated with ASD severity and the ability to inhibit involuntary attention.
Berman et al., 2016	*N* = 139 (95 ASD; 44 TD) *Ages*: 10.2 ASD; 10.4 TD	Examined auditory evoked latency (M100) and auditory mismatch field (MMF) in auditory cortex. Also examined structural integrity of white matter tracts associated with primary auditory processing and higher level auditory-linguistic processing.	*Passive (awake) task*: For the M100 task, tones were passively presented at 45 dB and repeated 130 times. For the MMF task, auditory stimuli (vowels /a/ and /u/) with deviant stimuli at randomized positions (15% probability) were presented with 300 ms duration.	↓ slower left hemisphere white matter maturation across ROIs in ASD. ↓ slower M100 latency maturation in ASD. ↓ later MMF in ASD with language impairment. Delayed M100 associated with ↑ ASD severity. Delayed MMF associated with ↑ language impairment. White matter structures contributed to M100 and MMF latency in TD, but not ASD.	Atypical development of white matter tracts and neural functioning in auditory and language systems in ASD. M100 and MMF latency were of clinical significance for ASD severity and language performance respectively.
Bloy et al., 2019	*N* = 50 (35 ASD; 15 TD) *Ages*: 6 to 10.9 yrs	Examined event related desynchronization (ERD) in the auditory cortex (theta—low beta band) in the late fields post stimulus (200–1000 ms). *Regions of interest*: right/left auditory cortex, and non-auditory regions (left/right frontal lobe, left/right parietal lobe, and mid sagittal locations [CM, FM, PM, OpM, FpM]).	*Passive (awake) task*: Stimuli consisted of words and plausible non-words. Auditory tokens were presented with a random inter-trial interval ranging from 5.0 to 5.2s. Stimuli from each class were randomly presented for a total scan time of 22 min.	↑ ERD associated with better language performance. ERD did not differ between groups. ERD was not correlated with ASD severity.	ERD is an index of language ability across groups and is not confounded by ASD.
Brennan et al., 2016	*N* = 25 (12 ASD- HF; 13 TD) *Ages*: 8–12 yrs *HF = high-functioning.	Examined evoked responses associated with phonological processing at 700 ms post-stimulus onset (330ms after target phoneme onset). Early processing of sounds (M100) was also included for replication. *Region of interest*: Left and right auditory cortex.	*Passive (awake) task*: Stimuli consisted of two items with a phonotactically legal consonant sequence and two with a phonotactically illegal sequence created by switching only the final two consonants. Each participant heard 24 repetitions of each word, yielding 48 phonotactically legal trials and 48 illegal trials.	↑ response to legal than illegal sequences from 733 to 816 ms (~ 330 ms post target) in right auditory cortex for ASD but no difference between legality for TD. ↑ response across sequence type from 433 to 531 ms (~ 180 ms post target) in left auditory cortex for ASD compared to TD. Delayed latency for right M100 in ASD.	Atypical phonological processing in ASD possibly due to differences in initial acoustic processing.
Demopoulos et al., 2023	*N* = 58 ASD *Ages*: 5 to 18 yrs	Examined rapid auditory processing of speech sounds via auditory evoked responses. *Regions of interest*: M100 (~ 100 ms) and 300–600 ms in auditory cortex.	*Passive (awake) task*: A Rapid Speech Sound Processing Task was performed to capture auditory evoked fields in response to hearing two different speech sounds (“ga” and “da”) presented in rapid succession.	No hemispheric difference in auditory processing. Bilateral processing of speech sounds was associated with speech articulation. Left hemisphere processing was associated with expressive language, expressive vocabulary, and phonological memory.	Atypical rapid auditory processing for speech sounds is associated with dysfunction in verbal communication in ASD group.
Kovelman et al., 2015	*N =* 19 (10 ASD; 9 TD) Ages: 6 to 12 yrs	Examined neural activity in temporal regions for syllable processing.	*Active task*: English-speaking participants were familiarized with an Italian passage. After familiarization, they listened to list of words that included target words from Italian passage and novel words. Participants decided for each word if it “sounded like it could be part of the new language”.	ASD had ↓ accuracy in extracting words from foreign speech. ASD displayed different brain activation during the learning phase *(no details on how it was different)*.	Efficiency with which left temporal regions process rhythmic information may be important for gains in language and reading proficiency.
Wagley et al., 2020	*N* = 29 (15 ASD [1 F, 14 M]; 14 TD [1 F, 13 F]). *Ages*: 8 to 12 yrs	Examined neural index of learning as increase in ERF between 200–500 ms post stimulus as function of surprise and repetition. *Regions of interest*: left A1, left pSTG, and left IFG.	*Passive (awake) task*: Participants listened to recordings of a non-native passage with grammatically plausible but semantically nonsensical sentences. Three repetitions of the same passage with focus on 8 key syllables. Children were measured for their learning of key syllables.	ASD ↓ accurate identification of target words (behavioral). TD showed ↑ response in left A1, left pSTG, and left IFG with surprisal and repetition (neural index of learning). ASD showed flat to negative trend with repetition and surprisal.	ASD showed lack of neural index of learning with impairments in using distributional cues (probabilities) to find words in speech.
Yau et al., 2015	*N* = 23 (1 ASD-NV [F]; 18 TD [M];13 ASD-V [M] *Ages*: 6 to 14 yrs *NV = nonverbal *V= verbal	Examined event-related responses at 50 ms (M50) and 100ms (M100) for auditory processing of speech and non-speech stimuli in auditory cortex. Follow-up EEG examined ERPs for auditory processing of tones.	Study 1 *passive (awake) task*: Participants listened to speech sounds and complex tones. Study 2 *passive (awake) task*: Participants listened to pure tone stimuli to examine if MEG results in study 1 were consistent.	TD and ASD showed similar responses to speech and non-speech. ↑ (larger and earlier) responses to non-speech than speech in non-verbal. Non-verbal showed double-peaked M50 and M100 response for non-speech and flat response to speech in auditory cortex. ↑ (larger and earlier) responses to pure tones at F3 (left frontal) and F4 (right frontal) electrodes via EEG	Non-verbal case study had atypically strong responses to non-speech and weak responses to speech. Highlighted the feasibility of MEG-EEG in minimally verbal participants.

*CM* cingulate motor area, *FM* frontal medial cortex, *FpM* frontopolar medial cortex, *IFG* inferior frontal gyrus, *MFG* middle frontal gyrus/premotor cortex, *MTG* middle temporal gyrus, *OpM* opercular motor area, *PM* premotor cortex, *A1* primary auditory cortex, *M1* primary motor cortex, *sSTG* uperior temporal gyrus, *SMG* supramarginal gyrus

#### Infancy and early childhood

Foundational language processing, including auditory and perceptual mechanisms, exhibits differences in infants with an elevated likelihood of autism, compared to those with a lower likelihood. EEG studies found atypical responses to repetitive speech (vowels), including slower mismatch negativity (MMN) latencies at 5 and 10 months old (Begum-Ali et al., 2021) and larger P150 amplitudes at 9 months old (Seery et al., 2014), suggesting slower neural discrimination and heightened attention allocation for speech stimuli, respectively. In alignment, fNIRS studies found reduced activation in anterior regions of the bilateral temporal lobe during perceptual processing of repetitive syllable sequences in infants aged 3–6 months (Edwards et al., 2017; Pecukonis et al., 2021). Similarly, fMRI studies found that elevated likelihood infants (4–9 months old) exhibited weaker bilateral activation to speech stimuli (e.g., human voice, speech versus nonspeech) in the temporal lobe, specifically in the superior temporal gyrus (STG; Blasi et al., 2015; Liu et al., 2021) and Heschl’s gyrus (Liu et al., 2021), possibly reflective of reduced sensitivity to human voices or less efficient auditory-phonological processing. Notably, when later language outcomes were considered, distinct neural activation patterns emerged. Infants (12 months) later diagnosed with autism with language impairment showed hypoactivation in the left STG, superior temporal sulcus (STS), supramarginal gyrus (SMG), and Heschl’s gyrus compared to non-autistic peers and autistic peers without language impairment (Lombardo et al., 2015). In contrast, infants later diagnosed with autism without language impairment did not differ from non-autistic peers in STS and Heschl’s gyrus activation. These findings suggest that early hypoactivation in temporal regions may not be specific to autism likelihood itself but could be linked to language impairments within autism.

Higher-order linguistic processing also shows differences in infants with an elevated likelihood of autism. EEG studies found atypical late ERP responses, including larger negative late slow wave (LSW) amplitudes in the left hemisphere for consonant-vowel streams in infants (12 months old) later diagnosed with autism (Finch et al., 2017), and larger N200 amplitudes for late-acquired words in elevated likelihood toddlers (3 years old) with language delays (Finch et al., 2018), suggestive of less efficient integration of speech-relevant information (i.e., increased effort). In alignment, an fMRI study found that elevated likelihood infants (4–7 months old) exhibited weaker bilateral activation to speech-like stimuli in the middle temporal gyrus (MTG), and superior and middle frontal gyri (SFG, MFG), possibly reflective of less efficient processing of linguistic information or reduced sensitivity to linguistic features (Blasi et al., 2015). Of note, infants (12 months) later diagnosed with autism with language impairment showed hypoactivation in the left MTG compared to non-autistic peers and autistic peers without language impairment (Lombardo et al., 2015). In contrast, infants later diagnosed with autism without language impairment did not differ from non-autistic peers in MTG activation, suggesting that hypoactivation in middle temporal regions may be linked to language impairments within autism.

#### Mid-to-late childhood

Similar to infancy, there are differences in auditory and perceptual language processing in both minimally speaking and speaking autistic children relative to non-autistic peers. EEG studies found atypical responses in early ERP components, including larger N1 amplitudes in speaking (4–11 years old; Chen et al., 2021) and minimally speaking autistic children (9.5–14.5 years old; Yu et al., 2023), opposite to non-autistic children who showed larger N1 amplitudes for speech compared to non-speech. These studies suggest an atypical prioritization of non-speech sounds in early auditory processing across language abilities in autism. Additionally, minimally and nonspeaking autistic children (3.5-8 years old) exhibited slower P1 latencies (Cantiani et al., 2016) and speaking autistic children (4–11 years old) exhibited weaker P1 amplitudes (Chen et al., 2021), suggesting slower neural detection and reduced sensitivity to speech. In alignment, MEG studies report that autistic children with varying language abilities (7–12 years old) exhibited delayed M100 latencies for speech stimuli in the right (Brennan et al., 2016) and bilateral auditory cortex (Berman et al., 2016), suggesting slower auditory processing for speech. On the other hand, fMRI studies found that autistic children and adolescents (5–18 years old) with varying language abilities did not differ from non-autistic children in left temporal lobe activation for various speech stimuli (e.g., syllables, sung words), specifically in the STG (O’Brien et al., 2023; Sharda et al., 2015), STS (Abrams et al., 2019; Sharda et al., 2015), and Heschl’s gyrus (Abrams et al., 2019), indicating typical leftward lateralization in childhood, possibly due to compensatory mechanisms.

Similar to infancy, higher-order linguistic processing shows differences in both minimally speaking and speaking autistic children. EEG studies found atypical late ERP responses, including decreased N400 amplitudes during semantic integration (Cantiani et al., 2016) and an absence of a left lateralized late negative response (LNR) for native words (Yu et al., 2023) in minimally speaking autistic children (3.5-8- and 9.5-14.5-year-olds, respectively). Speaking autistic children (4–6 years old) did not display a mismatch effect (N600/P600) when distinguishing non-speech from speech (Galilee et al., 2017). Together, these EEG studies suggest that autism may be associated with less efficient semantic and lexical integration across language abilities. In alignment, MEG studies found that speaking autistic children (8–12 years old) displayed reduced late responses to surprisal in the left auditory cortex (Wagley et al., 2020) and stronger late responses to speech that violates phonotactic rules in the right auditory cortex (Brennan et al., 2016), suggesting reduced sensitivity to linguistic predictions and atypical hemispheric lateralization for linguistic processing. Similarly, an fNIRS study found that non-autistic children displayed increasing left temporal activation as linguistic hierarchy increased (e.g., scrambled speech to native speech), whereas autistic children (3–10 years old) lacked leftward asymmetry across linguistic hierarchies, suggesting altered sensitivity to linguistic information (Lai et al., 2024). Furthermore, an fMRI study found that autistic children (6–16 years old) exhibited weaker activation in the left inferior frontal gyrus (IFG) for spoken words, but not for sung words, possibly reflective of reduced sensitivity to linguistic features (Sharda et al., 2015).

## Discussion

This review systematically synthesized 31 functional neuroimaging studies on language development in autistic children, aiming to identify patterns and cross-modal connections that previous reviews may have missed. When evaluating neural differences in autism, it is critical to consider the trajectory of language development in typically developing children, which is well-established from neuroimaging studies. Typically developing infants orient to human voices from birth and display increased bilateral temporal activation to human voices compared to environmental sounds (Huberty et al., 2023; Kuhl, 2010). In the present review, infants with an elevated likelihood of autism demonstrate weaker activation to human voices in the temporal lobe (Blasi et al., 2015), possibly reflective of reduced attentional allocation to human voices. Between 9 and 12 months, typically developing infants can distinguish between speech and nonspeech sounds with increased bilateral activation in frontal and temporal lobes for speech stimuli (Vouloumanos & Gelfand, 2013). In the present review, fMRI and fNIRS studies found that elevated likelihood infants displayed weaker bilateral activation in the temporal lobes for speech-like stimuli (Edwards et al., 2017; Liu et al., 2021; Pecukonis et al., 2021), along with delayed and larger early ERP components for speech (Begum-Ali et al., 2021; Seery et al., 2014). Overall, these findings indicate that atypical patterns of perceptual speech processing begin to manifest within the first year of life for infants with an increased likelihood of autism and could indicate slower neural detection and/or reduced sensitivity to speech.

In typical development, language processing shifts from broad, bilateral hemispheric activation to left lateralized activation (around ages 5–10 years) in specific structures, including the left IFG, MFG, STS, and STG (Friederici, 2011; Holland et al., 2007; Kuhl, 2010). In this review, most fMRI studies did not find differences between autistic and non-autistic children (5–18 years old; varying language abilities) in left temporal activation for speech, including in the STG and STS, indicative of typical leftward activation possibly due to compensatory mechanisms (Abrams et al., 2019; O’Brien et al., 2023; Sharda et al., 2015). On the other hand, an fNIRS study found that autistic children (3–10 years old) lacked leftward lateralization in temporal regions (Lai et al., 2024), and Sharda et al., (2015) found that autistic children (6–16 years old) exhibited weaker activation in the left IFG, indicative of atypical lateralization. Previous fMRI-focused literature reviews report both reduced left lateralization and reversed right lateralization across frontal and temporal regions (Cermak et al., 2022; Herringshaw et al., 2016; Mody & Belliveau, 2013; Sperdin & Schaer, 2016); the present review highlights that atypical temporal lateralization is not always seen in autistic children, possibly due to the heterogeneity of language itself, compensatory neural mechanisms, or due to the variability in experimental tasks and participant ages.

In typically developing children, high-order linguistic processes, including semantic and syntactic processing, emerge in frontotemporal networks during early childhood and continue maturing as language skills develop (Brauer & Friederici, 2007; Klein et al., 2023). EEG studies show that typically developing children (from 3 to 4 years old) display the N400 component associated with semantic processing and slightly older children (from 5 to 6 years old) display the P600 component associated with syntactic processing (Silva-Pereyra et al., 2007; Wang et al., 2020). In the present review, elevated likelihood infants displayed larger LSW and N200 amplitudes (Finch et al., 2017, 2018) and autistic children (3.5–14.5 years old) of varying language abilities displayed weaker N400 amplitudes and lacked a P600 mismatch effect (Cantiani et al., 2016; Galilee et al., 2017). Taken together, these studies suggest that autism may be associated with less efficient integration and reduced sensitivity for linguistic information across developmental stages. Overall, although atypical left lateralization and hypoactivation in temporal regions were not consistently seen in the present review for mid-to-late childhood studies, the timing and magnitude of auditory, perceptual, and higher-order linguistic processing within fronto-temporal regions remained atypical from infancy to late childhood.

Although we synthesized cross-modal findings relative to developmental stage, it is important to note that participant ages varied within developmental stages. The reviewed studies indicate that atypical auditory, perceptual, and high-order linguistic processes associated with autism likelihood emerge within the first year of life. However, the variability in ages (e.g., 3 months to 12 months) prevents us from identifying a precise age at which neural differences first emerge. Moreover, there was also considerable variability in age in mid-to-late childhood studies; some studies included narrow age ranges (e.g., 4–6 years; Galilee et al., 2017), while others used broad ranges spanning from mid childhood to late adolescence (e.g., 6–16 years in Sharda et al., 2015; 8–21 years in Larson et al., 2022). Such variability could contribute to inconsistencies in neural findings between studies and make it difficult to understand the precise timing of when neural differences occur in autism. For example, infant studies found hypoactivation in the temporal lobe, but this was not consistently seen in mid-to-late childhood studies. Due to the cross-sectional design of the studies and variability in ages, it is difficult to conclude when temporal hypoactivation might change. Given that language follows an age-related trajectory, we strongly encourage future research to explore longitudinal changes from infancy into mid-childhood.

Beyond age, we examined other participant characteristics that are important considerations when interpreting findings. Consistent with previous findings *(*Jack & Pelphrey, 2017; Mody & Belliveau, 2013; Redcay & Courchesne, 2008*)*, we found a large bias towards autistic participants with average or above-average standardized intelligence, cognitive functioning, and verbal language abilities. Autistic children with intellectual disabilities and/or complex communication needs (i.e., minimally or nonspeaking) were underrepresented across studies. Importantly, in the limited studies that did include varying language abilities, autistic participants without language impairments sometimes did not differ from non-autistic participants, whereas autistic participants with language impairments differed from their autistic peers (e.g., Lombardo et al., 2015; Yu et al., 2023). Consequently, the heterogeneity of language within autism is an important factor in interpreting neural differences and warrants caution when generalizing findings from studies that only include speaking autistic children. We strongly urge researchers to include autistic children with varying language abilities, cognitive functioning, and autism traits to better elucidate the neural development of language in autism *(*Jack & Pelphrey, 2017*)*.

Furthermore, while most studies reported gender/sex in alignment with current prevalence estimates, gender/sex was only included in the data analysis in three studies (Edwards et al., 2017; Finch et al., 2018; Tran et al., 2021). Given the emerging evidence that autism may present differently by gender (Tsirgiotis et al., 2024), gender may contribute to variance in neural findings. For example, when sex was considered, 3-month-old elevated likelihood females, not males, showed increased activation to repetitive speech in the bilateral temporal lobe, suggesting a potential gender- and risk-specific endophenotype (Edwards et al., 2017). Consequently, we strongly urge researchers to include gender and/or sex as a factor in data analysis, accounting for potential effects when determining sample size. Similarly, only eight studies provided ethnic, racial, and/or socioeconomic compositions of their samples; participants were predominantly white and had higher socioeconomic statuses. Language and communication in autism can manifest differently across cultural and racial groups (de Leeuw et al., 2020; Mandell et al., 2009), limiting the generalizability of findings from predominantly white participants. Additionally, socioeconomic status contributes to disparities in access to diagnostic and support services for autistic children (Bishop-Fitzpatrick & Kind, 2017), which was rarely accounted for in the included studies. We strongly encourage researchers to include participants of diverse ethnic, racial, and socioeconomic backgrounds to better understand the neural development of language in autism.

Regarding experimental design, most of the studies used passive awake tasks, where participants listened to stimuli while engaging in unrelated activities (e.g., Abrams et al., 2019; Dunham-Carr et al., 2023), and a handful of studies used active tasks in which participants responded to the stimuli (e.g., Larson et al., 2022; O’Brien et al., 2023). Passive tasks may enable studies in younger and minimally speaking participants; however, passive and active tasks differ in their cognitive demands, subsequently impacting neural activity (Ioannucci et al., 2023). Other reviews highlight this, with autistic children showing atypical processing during a passive auditory task but not in an active auditory task, emphasizing that findings cannot be generalized across task modes (Marco et al., 2011; Whitehouse & Bishop, 2008). Additionally, within passive tasks, infant fMRI studies in the present review were conducted during natural sleep to reduce motion artifacts (Blasi et al., 2015; Liu et al., 2021; Lombardo et al., 2015), whereas infant EEG and fNIRS studies were conducted while awake (e.g., Edwards et al., 2017; Seery et al., 2014; Tran et al., 2021). Although passive auditory tasks can induce neural activity during sleep, the magnitude of activation and functional connectivity can differ between behavioral states (Yates et al., 2023). Consequently, task mode (i.e., passive or active) and behavioral state (i.e., asleep or awake) could contribute to the variance in neural findings between the studies. We caution the generalizability of passive and/or sleep studies to naturalistic settings where active engagement with language is often required. More robust research using multiple task modes and behavioral states is needed to further understand the neural development of language in autism.

Furthermore, across active and passive tasks, there was variance in the complexity of speech stimuli used; around half of the studies used simple stimuli (e.g., speech sounds, syllables) while the other half used more complex stimuli, including words and/or sentences. Language processing is hierarchical, with neural activity differing depending on the complexity of the speech stimuli; simple stimuli activate auditory and midfrontal regions, whereas complex stimuli recruit broader frontotemporal networks (Gernsbacher & Kaschak, 2003; Rimmele et al., 2023). Consequently, the variability in speech stimuli used across the studies in the present review likely contributes to variance in results. Moreover, only a few studies provided information about participants’ familiarity with the stimuli used. The level of familiarity with speech stimuli can modulate neural activity, with familiar words typically eliciting stronger responses and interhemispheric connectivity (Kim et al., 2022; Leech et al., 2009; Ning et al., 2018). In the limited studies that detailed participant familiarity, familiarity with the words modulated neural responses in autistic children (Finch et al., 2018; Sandbank et al., 2017). Most of the studies did not mention familiarity; therefore, it is possible that familiarity impacted neural responses. In alignment with Sandbank et al. (2017), we highlight the importance of tracking stimuli familiarity in future research.

The reviewed studies met most quality indicators; however, of particular interest is sample size justification, which was only met by two studies (Begum-Ali et al., 2021; Dunham-Carr et al., 2023). Similar to our review, Szucs and Ioannidis (2020*)* found that only 3% of neuroimaging studies performed power calculations, and they urged researchers to conduct pre-study power calculations. Sample size is critically important to account for the natural variation in neural activity between individuals (Szucs & Ioannidis, 2020; Turner et al., 2018). In the reviewed studies, autistic groups had a median sample size of 22 participants, and non-autistic groups had a median of 20, with over half of the studies not reaching the recommended 25 participants per group (Szucs & Ioannidis, 2020). The sample size of the included studies is concerning, especially given how heterogeneous language is within autism. In supporting recent calls, we urge researchers to conduct and report power analyses and account for language heterogeneity during participant recruitment.

## Conclusion

Understanding the neurobiological trajectory of language in autism is critical for advancing individualized support services. Overall, we found that infants with an elevated likelihood of autism displayed hypoactivation in bilateral temporal regions in response to speech stimuli, along with delayed timing and larger amplitudes in both auditory-perceptual and higher-order linguistic processing. There were distinct patterns for infants who were later diagnosed with language impairments, which urges caution when generalizing findings across all elevated likelihood infants. In contrast to other reviews, we did not find consistent hypoactivation in left temporal regions in autistic children; however, the timing and magnitude of auditory-perceptual and higher-order linguistic processing remained atypical from infancy to late childhood. Although the breadth of research conducted across functional neuroimaging modalities is promising, we found a lack of replication and consistency across neural components evaluated, considerable variability in participant ages, and minimal participant diversity. Consequently, we urge researchers to further explore how diverse autistic individuals perceive, process, and produce language to better understand the neurobiological trajectory of language and to improve the translation of neuroimaging findings (Wilkinson et al., 2015). Future research should include autistic participants with diverse autism traits, language abilities, gender identities, ethnic/racial identities, and socioeconomic statuses (Mody & Belliveau, 2013).

## Supplementary Information

Below is the link to the electronic supplementary material.

Supplementary Material 1 (DOCX 29.2 KB)

## Data Availability

No datasets were generated or analysed during the current study.
