# Supplementary material for: Neurobiology of language in autism: a systematic review of pediatric studies
Source: Brain Imaging Behav. 2026 Mar 25;20(2):60. doi: 10.1007/s11682-026-01140-y (PMC13018016; doi:10.1007/s11682-026-01140-y)
Supplement: Supplementary file 1 — Supplementary Material 1 (DOCX 29.2 KB) [file 11682_2026_1140_MOESM1_ESM.docx]

**Supplementary Table 1**

*Results of AXIS quality assessment*

|  | Introduction | Methods | | | | | | | | | | Results | | | | | Discussion | | Other | |
| --- | --- | --- | --- | --- | --- | --- | --- | --- | --- | --- | --- | --- | --- | --- | --- | --- | --- | --- | --- | --- |
| **Article** | *Q1* | *Q2* | *Q3* | *Q4* | *Q5* | *Q6* | *Q7* | *Q8* | *Q9* | *Q10* | *Q11* | *Q12* | *Q13* | *Q14* | *Q15* | *Q16* | *Q17* | *Q18* | *Q19* | *Q20* |
| Abrams et al., 2019 | Y | Y | N | Y | Y | NC | Y | Y | Y | Y | Y | Y | N | Y | NC | Y | NC | Y | N | Y |
| Alho et al., 2023 | Y | Y | N | Y | Y | NC | Y | Y | Y | Y | Y | Y | N | Y | NC | Y | Y | Y | N | Y |
| Begun-Ali et al., 2021 | Y | Y | Y | Y | Y | Y | Y | Y | Y | Y | Y | Y | Y | Y | NC | Y | Y | NC | N | Y |
| Berman et al., 2016 | Y | Y | N | Y | Y | Y | N | Y | Y | Y | Y | Y | NC | N | NC | Y | Y | Y | N | Y |
| Blasi et al., 2015 | Y | Y | N | Y | Y | Y | Y | Y | Y | NC | Y | NC | N | Y | NC | NC | Y | Y | N | Y |
| Bloy et al., 2019 | Y | Y | N | Y | Y | NC | Y | Y | Y | Y | Y | Y | Y | Y | NC | Y | Y | N | N | Y |
| Brennan et al., 2016 | Y | Y | N | Y | Y | Y | Y | Y | Y | Y | Y | Y | N | Y | NC | Y | NC | NC | N | NC |
| Cantiani et al., 2016 | Y | Y | N | Y | Y | Y | Y | Y | Y | Y | Y | Y | N | Y | NC | Y | Y | Y | N | Y |
| Chen et al., 2021 | Y | Y | N | Y | Y | Y | Y | Y | Y | Y | Y | Y | N | Y | Y | Y | Y | Y | N | Y |
| Demopoulos et al., 2023 | Y | Y | N | Y | Y | Y | Y | Y | Y | Y | Y | Y | N | Y | Y | Y | Y | Y | N | Y |
| Dunham-Carr et al., 2023 | Y | Y | Y | Y | Y | Y | N | Y | Y | Y | Y | Y | NC | N | NC | Y | NC | Y | Y | Y |
| Edwards et al., 2017 | Y | Y | N | Y | Y | Y | Y | Y | Y | Y | Y | Y | N | Y | NC | Y | Y | Y | N | Y |
| Eigsti et al., 2016 | Y | Y | N | Y | Y | Y | N | Y | Y | Y | Y | Y | NC | N | NC | Y | Y | Y | N | Y |
| Finch et al., 2017 | Y | Y | N | Y | Y | Y | Y | Y | Y | Y | Y | Y | N | Y | NC | Y | Y | Y | N | Y |
| Finch et al., 2018 | Y | Y | N | Y | Y | Y | Y | Y | Y | Y | Y | Y | N | Y | NC | Y | Y | Y | N | Y |
| Galilee et al., 2017 | Y | Y | N | Y | Y | Y | Y | Y | Y | Y | Y | Y | N | Y | NC | Y | Y | Y | N | Y |
| Green et al., 2020 | Y | Y | N | Y | Y | Y | N | Y | Y | Y | Y | Y | Y | N | NC | Y | Y | Y | N | Y |
| Kovelman et al., 2015 | Y | Y | N | NC | Y | NC | N | NC | NC | N | N | N | NC | N | NC | N | NC | N | NC | NC |
| Lai et al., 2024 | Y | Y | N | Y | Y | Y | Y | Y | Y | Y | Y | Y | Y | Y | NC | Y | Y | Y | N | Y |
| Larson et al., 2022 | Y | Y | N | Y | Y | Y | Y | Y | Y | Y | Y | Y | N | Y | NC | Y | Y | Y | N | NC |
| Liu et al., 2021 | Y | Y | N | Y | Y | Y | Y | Y | Y | NC | Y | Y | N | Y | NC | Y | Y | Y | N | Y |
| Lombardo et al., 2015 | Y | Y | N | Y | Y | NC | Y | Y | Y | Y | Y | Y | NC | NC | NC | Y | Y | N | N | Y |
| O'Brien et al., 2023 | Y | Y | N | Y | Y | Y | Y | Y | Y | Y | Y | Y | N | Y | NC | Y | Y | Y | N | Y |
| Pecukonis et al., 2021 | Y | Y | N | Y | Y | Y | Y | Y | Y | Y | Y | Y | Y | Y | NC | Y | Y | Y | N | Y |

^1^ Codes for quality rating indicators are as follows: Y = yes; N = no; NC = not clear or not known. If the answer to questions 13 and/or 19 is “No”, this is considered meeting the quality indicator.

*(Supp Table 1 Cont.)*

|  | Introduction | Methods | | | | | | | | | | Results | | | | | Discussion | | Other | |
| --- | --- | --- | --- | --- | --- | --- | --- | --- | --- | --- | --- | --- | --- | --- | --- | --- | --- | --- | --- | --- |
| **Article** | *Q1* | *Q2* | *Q3* | *Q4* | *Q5* | *Q6* | *Q7* | *Q8* | *Q9* | *Q10* | *Q11* | *Q12* | *Q13* | *Q14* | *Q15* | *Q16* | *Q17* | *Q18* | *Q19* | *Q20* |
| Sandbank et al., 2017 | Y | Y | N | Y | Y | Y | Y | Y | Y | Y | Y | Y | NC | Y | NC | Y | Y | Y | N | Y |
| Seery et al., 2014 | Y | Y | N | Y | Y | Y | Y | Y | Y | Y | Y | Y | Y | Y | NC | Y | Y | Y | N | Y |
| Sharda et al., 2015 | Y | Y | N | Y | Y | Y | N | Y | Y | Y | Y | NC | N | N | NC | Y | Y | NC | N | Y |
| Tran et al., 2021 | Y | Y | N | Y | Y | Y | N | Y | Y | Y | Y | Y | N | N | NC | Y | Y | Y | N | Y |
| Wagley et al., 2020 | Y | Y | N | Y | Y | Y | Y | Y | Y | Y | Y | Y | N | Y | NC | Y | Y | Y | NC | Y |
| Yau et al., 2015 | NC | Y | N | Y | Y | Y | N | Y | Y | NC | Y | NC | NC | N | NC | NC | Y | N | N | Y |
| Yu et al., 2023 | Y | Y | N | Y | Y | Y | N | Y | Y | Y | Y | Y | NC | N | NC | Y | Y | Y | N | NC |
